# Exploring small-scale chemostats to scale up microbial processes: 3-hydroxypropionic acid production in *S. cerevisiae*

**DOI:** 10.1186/s12934-019-1101-5

**Published:** 2019-03-11

**Authors:** Alicia V. Lis, Konstantin Schneider, Jost Weber, Jay D. Keasling, Michael Krogh Jensen, Tobias Klein

**Affiliations:** 1 The Novo Nordisk Foundation Center for Biosustainability, Technical University of Denmark, Kongens Lyngby, Denmark; 2 Present Address: BASF SE, Ludwigshafen am Rhein, Germany; 3 Present Address: CureVac AG, Tübingen, Germany; 4 Joint BioEnergy Institute, Emeryville, CA USA; 5 Biological Systems & Engineering Division, Lawrence Berkeley National Laboratory, Berkeley, CA USA; 6 Department of Chemical and Biomolecular Engineering, University of California, Berkeley, CA USA; 7 Department of Bioengineering, University of California, Berkeley, CA USA

**Keywords:** 3-HP, Small-scale chemostat, Fed-batch, *S. cerevisiae*, Substrate limitation

## Abstract

**Background:**

The physiological characterization of microorganisms provides valuable information for bioprocess development. Chemostat cultivations are a powerful tool for this purpose, as they allow defined changes to one single parameter at a time, which is most commonly the growth rate. The subsequent establishment of a steady state then permits constant variables enabling the acquisition of reproducible data sets for comparing microbial performance under different conditions. We performed physiological characterizations of a 3-hydroxypropionic acid (3-HP) producing *Saccharomyces cerevisiae* strain in a miniaturized and parallelized chemostat cultivation system. The physiological conditions under investigation were various growth rates controlled by different nutrient limitations (C, N, P). Based on the cultivation parameters obtained subsequent fed-batch cultivations were designed.

**Results:**

We report technical advancements of a small-scale chemostat cultivation system and its applicability for reliable strain screening under different physiological conditions, i.e. varying dilution rates and different substrate limitations (C, N, P). Exploring the performance of an engineered 3-HP producing *S. cerevisiae* strain under carbon-limiting conditions revealed the highest 3-HP yields per substrate and biomass of 16.6 %C-mol and 0.43 g gCDW^−1^, respectively, at the lowest set dilution rate of 0.04 h^−1^. 3-HP production was further optimized by applying N- and P-limiting conditions, which resulted in a further increase in 3-HP yields revealing values of 21.1 %C-mol and 0.50 g gCDW^−1^ under phosphate-limiting conditions. The corresponding parameters favoring an increased 3-HP production, i.e. dilution rate as well as C- and P-limiting conditions, were transferred from the small-scale chemostat cultivation system to 1-L bench-top fermenters operating in fed-batch conditions, revealing 3-HP yields of 15.9 %C-mol and 0.45 g gCDW^−1^ under C-limiting, as well as 25.6 %C-mol and 0.50 g gCDW^−1^ under phosphate-limiting conditions.

**Conclusions:**

Small-scale chemostat cultures are well suited for the physiological characterization of microorganisms, particularly for investigating the effect of changing cultivation parameters on microbial performance. In our study, optimal conditions for 3-HP production comprised (i) a low dilution rate of 0.04 h^−1^ under carbon-limiting conditions and (ii) the use of phosphate-limiting conditions. Similar 3-HP yields were achieved in chemostat and fed-batch cultures under both C- and P-limiting conditions proving the growth rate as robust parameter for process transfer and thus the small-scale chemostat system as powerful tool for process optimization.

## Introduction

Besides strain engineering, the establishment of a commercially viable bioprocess requires multiple screening procedures, process development, and optimizations as well as process validation and scale-up trials. Fully equipped stirred tank reactors are considered to be the gold standard for quantitative strain characterizations, allowing versatile cultivation modes, such as batch and fed-batch, and the precise on-line measurement and control of various cultivation parameters, such as dissolved oxygen and pH levels. Novel microbioreactor (MBR) systems, ranging from down-scaled stirred tank reactors to advanced shaken microtiter plate cultivation devices, have been developed to meet the increasing need for high throughput strain screening and testing of relevant cultivation conditions [1]. Efforts towards designing MBR systems are reflected in robust cultivation devices, such as the parallelized small-scale chemostat bioreactor system based on Hungate tubes [2] and the microtiter plate based system for high-throughput temperature optimization for microbial and enzymatic systems [3]. A few MBR systems have been further developed, and even reached commercialization, such as the *ambr* system (Sartorius) or the *BioLector* (m2p-labs). Certain MBR systems allow various cultivation modes with the possibility to monitor and control process parameters selectively, whereas other systems only support one mode of cultivation, i.e. continuous or fed-batch mode. Chemostat cultivations provide defined and constant cultivation conditions, where single parameters such as temperature, pH, nutrient composition or concentration can be investigated in relation to the growth rate applied [4]. Industrial bioprocesses usually favor cultivations in fed-batch mode, as this cultivation mode has been found effective in circumventing undesired phenomena, such as substrate inhibition, catabolite repression or the Crabtree effect [5, 6]. Fed-batch cultivations are characterized by feeding nutrients intermittently or continuously for microbial growth. The specific growth rate in fed-batch cultivations can be controlled by feeding a single growth-limiting nutrient at a desired rate. As both chemostat and fed-batch cultivations are capable of tightly maintaining the specific growth rate at a set value without the accumulation of residual substrate, key cultivation parameters obtained in chemostat and fed-batch cultivations can be compared [7].

In this study, chemostat and fed-batch cultivations were performed with a *Saccharomyces cerevisiae* strain that has been genetically modified to express the biosynthetic pathway for 3-hydroxypropionic acid (3-HP, C_3_H_6_O_3_) via the intermediate β-alanine [8]. We used an improved small-scale parallelized continuous cultivation system to evaluate microbial performance for 3-HP production at different growth rates under carbon-limiting conditions. The best growth rate was subsequently applied to elucidate the effect of nitrogen- and phosphate-limiting conditions on product yield. The parameters assessed in small-scale chemostats were transferred to bench-top fed-batch cultivations and the microbial performance regarding 3-HP production was compared.

## Materials and methods

### Strains and maintenance

All cultivation experiments were performed with the genetically engineered *S. cerevisiae* strain ST938, previously described for 3-HP production by Borodina et al. [8]. The genes encoding the following enzymes were overexpressed in this strain to enable 3-HP production from aspartate via β-alanine: pyruvate carboxylase (*PYC1*, *PYC2*), aspartate aminotransferase (*AAT2*), aspartate decarboxylase (*TcPAND*), β-alanine-pyruvate aminotransferase (*BcBAPAT*), and 3-hydroxypropanoate dehydrogenase (*EcYDFG*). Biomass from the *S. cerevisiae* ST938 glycerol stock was distributed on a YPD agar plate and incubated for 3 days at 30 °C. For pre-culture preparations, a single colony from plate was used to inoculate 25 mL of synthetic medium in a 250-mL baffled shake flask. The culture was incubated in an orbital shaker at 30 °C degree and 250 rpm with a 2.5 cm orbit for approximately 24 h.

### Media

Synthetic medium consisted of 7.5 g L^−1^ (NH_4_)_2_SO_4_, 14.4 g L^−1^ KH_2_PO_4_, 0.5 g L^−1^ MgSO_4_·7H_2_O and 2% glucose with the addition of 2 mL trace metal and 1 mL vitamin solution. The trace metal solution (pH 6.0) comprised 4.5 g L^−1^ CaCl_2_·2H_2_O, 4.5 g L^−1^ ZnSO_4_·7H_2_O, 3 g L^−1^ FeSO_4_·7H_2_O, 1 g L^−1^ H_3_BO_3_, 1 g L^−1^ MnCl_2_·4H_2_O, 0.4 g L^−1^ Na_2_MoO_4_·2H_2_O, 0.3 g L^−1^ CoCl_2_·6H_2_O, 0.1 g L^−1^ CuSO_4_·5H_2_O, 0.1 g L^−1^ KI and 15 g L^−1^ EDTA. The vitamin solution (pH 6.5) consisted of 50 mg L^−1^ biotin, 200 mg L^−1^ *p*-aminobenzoic acid, 1 g L^−1^ nicotinic acid, 1 g L^−1^ Ca-pantothenate, 1 g L^−1^ pyridoxine-HCl, 1 g L^−1^ thiamine-HCl and 25 g L^−1^ myo-inositol. All chemicals were obtained from Sigma-Aldrich. The pH of the medium was adjusted to 5.0 and the medium afterwards sterile filtrated.

### Cultivations in small-scale continuous bioreactors

**Fig. 1:**
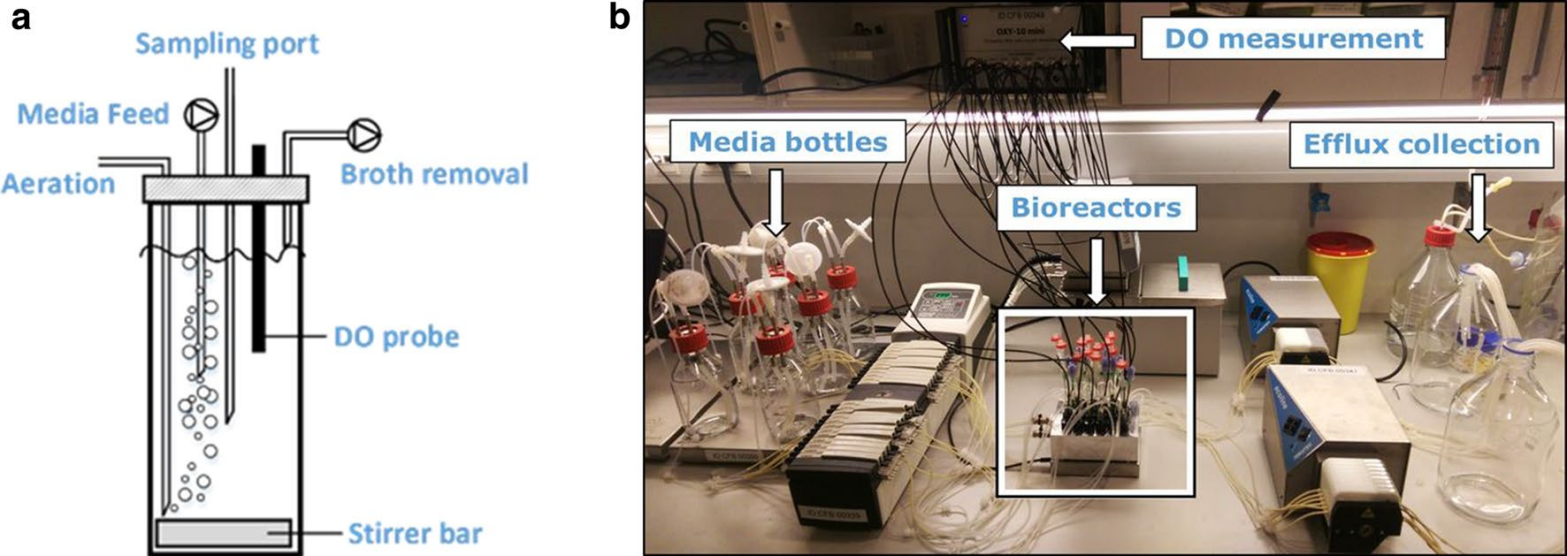
**a** Schematic view of a single stirred small-scale bioreactor for parallelized continuous cultivation. **b** Set-up of the parallel small-scale cultivation system

The custom-made small-scale continuous cultivation system consisted of 24 glass reactors built of 10-mL standard screw cap sample vials (VWR, Radnor, Pennsylvania, United States) that were placed in an aluminum block connected to a water bath (Julabo, Seelbach, Germany) for temperature control (Fig. 1). Stirring at the bottom of the bioreactors was carried out at 1000 rpm by using a 12 mm diameter triangle stirrer bar (VWR, Radnor, Pennsylvania, United States) and a microplate stirrer (MIXdrive 24 MTP, 2mag, Munich, Germany) for agitation. Besides the four ports for sampling, the lid housed ports for aeration, feed and broth removal, and an integrated glass tube with an immobilized fluorescence dye for dissolved oxygen (DO) measurements. DO was measured through polymer optical fibers (Presens, Regensburg, Germany), transferring excitation light to the sensor and the sensor response back to the multi-channel fiber optic oxygen meter (OXY-10 mini, Presens, Regensburg, Germany). A 24-channel peristaltic pump was used to supply fresh medium to the reactors (205U multichannel cassette pump with CA8 pump head, Watson-Marlow Pumps Group, Rommerskirchen, Germany). In order to achieve different dilution rates at fixed pump rates, tubes with different inner diameters were incorporated into the system (Marprene tubing: 0.25, 0.38, 0.50, 0.63 mm; Watson-Marlow, Rommerskirchen, Germany). A second microprocessor-controlled tubing pump (Ismatec EcoLine VC-ground unit with cassette head MS/CA 8-6, Ismatec Labortechnik GmbH, Wertheim, Germany) was connected to the medium outlet-port of the lid, operating at higher flow rates compared to the media pump to generate under pressure for passively aerating the system with humidified air to prevent evaporation from the cultivation system. For C-limited cultivation in the small-scale continuous bioreactors, the medium composition was as follows: 2 g L^−1^ (NH_4_)_2_SO_4_, 7.5 g L^−1^ glucose monohydrate, 3 g L^−1^ KH_2_PO_4_, 20 g L^−1^ 2-(*N*-morpholino)ethanesulfonic acid (MES), 0.5 g L^−1^ MgSO_4_·7H_2_O, with the addition of 2 mL trace metal and 1 mL vitamin solution, as described above. For N-limited cultivations the same medium composition was used with the exception that the glucose monohydrate concentration was 10 g L^−1^ and the ammonium sulfate ((NH_4_)_2_SO_4_) concentration was 0.51 g L^−1^. Correspondingly, P-limited conditions were achieved by applying 10 g L^−1^ glucose monohydrate and a potassium dihydrogen phosphate (KH_2_PO_4_) concentration of 0.08 g L^−1^. The pH of each medium was adjusted to 6.0 via addition of 2 M sodium hydroxide (NaOH), whereafter the medium was sterile filtrated. Each medium further contained 100 μL L^−1^ Antifoam 204 (Sigma-Aldrich, St. Louis, Missouri, United States). Inoculation of the continuous cultures through the sample port were performed with a syringe. The inoculum was 1 mL of a freshly grown *S. cerevisiae* strain ST938 culture adjusted to a cell density of 1 (OD_600nm_).

### Cultivations in 1-L bench-top bioreactors

Fed-batch fermentation were carried out in 1-L Biostat^®^ Qplus bench-top reactors (Sartorius, Goettingen, Germany). The cultivation regime comprised an initial batch phase, using 400 mL medium, followed by a second C- or P-limited feeding phase, adding another 400 mL of medium. The composition of the batch medium for C-limiting conditions was prepared as described above under section ‘medium’. For P-limiting conditions the amount of potassium dihydrogen phosphate (KH_2_PO_4_) was reduced to 0.04 g L^−1^. The reactors were equipped with pH, temperature and DO probes. The temperature was set to 30 °C and the pH was maintained at 5.5 by means of base addition (2 N NaOH). Oxygen limitation during the cultivation was prevented by keeping dissolved oxygen levels above 20% air saturation, which was assured by increasing the agitation speed up to a maximum of 1000 rpm and then by mixing the inlet air with pure oxygen if needed. The percentage of oxygen added to the air mix was automatically controlled by the process control software as response to the DO measurements.

The feed was started 1 h after carbon or phosphorus source depletion, indicated by decreasing CO_2_ levels in the off-gas and an increase in pH. The growth rate of the exponential feed profile was set to 0.05 h^−1^. We used an exponential feed profile according to the following equation:

$$F = a*\exp^{{\left( {\mu *t} \right)}}$$

with F = feed rate (g h^−1^), a = initial feed rate (g h^−1^), µ = specific growth rate of 0.05 h^−1^, and t = process time (h) from feed onset. The process was controlled by a commercial SCADA software (Lucullus PIMS, Securecell AG, Switzerland). The supply of the feed was controlled by means of gravimetric flow controllers. The feed for C-limiting conditions consisted of 400 g L^−1^ glucose monohydrate, 10 g L^−1^ MgSO_4_·7H_2_O, 30 g L^−1^ KH_2_PO_4_ as well as 6 mL trace element and 3 mL vitamin solution. The feed for P-limiting conditions was prepared analogously to the feed for C-limiting conditions with the exception of 3.2 g L^−1^ KH_2_PO_4_. Each feed solution further contained 100 μL L^−1^ Antifoam 204 (Sigma-Aldrich, St. Louis, Missouri, United States). All cultivations were carried out in triplicates.

### Determination of cell concentration and biomass dry weight

The optical density (OD_600nm_) of culture samples was measured in duplicate using a spectrophotometer (UV-1600PC Spectrophotometer, VWR, Radnor, Pennsylvania, United States) at a wavelength of 600 nm. Exact sample dilutions in water were determined gravimetrically. Biomass dry weight measurements were performed in duplicates by preparing different dilutions of a grown batch-culture in 15 mL-glass tubes. The respective cultures are measured for OD_600nm_, then washed and afterwards placed in an incubator at 60 °C for drying. The weight of the dried cell mass was determined. The calculated conversion factor for strain *S. cerevisiae* ST938 was 0.423 g L^−1^ per OD-unit at 660 nm.

### Analysis of extracellular metabolites

The extracellular concentrations of glucose, 3-HP, glycerol and ethanol were quantified by HPLC as previously described [8]. All compounds used as standards for calibration were obtained from Sigma-Aldrich, except 3-HP which was purchased from Tokyo Chemicals Industry Co.

Sample preparation for HPLC analysis involved the separation of the culture supernatant from the cells through centrifugation of the samples for 5 min at 13,000 rpm and 4 °C, followed by filtration (0.2 µm single-use syringe filter unit, Th. Geyer, Renningen, Germany) and 10× dilution in distilled water.

## Results and discussion

### Improved small-scale continuous cultivation system

In this study, we show further advancements and application possibilities of a small-scale continuous cultivation system, previously developed by Klein et al. [2], for an increased degree of parallelization and improved handling as well as monitoring of the individual reactors. The key aspects of the changes made to the system comprise an increase in the set of parallel culture vessels from 8 to 24 reactors and a decrease in working volume from 10 to 6.5 mL. The present system further consists of custom-made lids housing four fixed ports used for aeration, media supply, broth removal as well as inoculation or sampling (Fig. 1). Besides the four ports, an optical rod-shaped DO probe is inserted through the lid for DO monitoring without disturbing the cultivation process, and in this way replacing the oxygen fluorescent sensor spot of the previous set-up [2]. The water bath, which in the previous set-up maintained a constant cultivation temperature, was replaced by a custom-made aluminum heating block, which is fused to a microplate stirrer unit. As the previous version of the small-scale bioreactor system was validated using the fission yeast *Schizosaccharomyces pombe* [2], we here present the improved cultivation set-up for *S. cerevisiae* cultivations.

Basic operational steps, as well as adjustments of dilution rates by selecting the appropriate tube diameter and pump rate of the media influx pump, were performed as previously described [2]. Here, the weight of the liquid content of each bioreactor was determined gravimetrically at the end of the cultivation, allowing the precise calculation of the respective dilution rate with a 5.1% deviation. Culture broth and the reactor gas phase were both removed through the same port of the reactor lid using the efflux pump (Fig. 1). Efflux pump rates of 7.5 mL min^−1^ were used for all cultivation experiments. The efflux pump rate was far in excess of the feeding pump rate, generating a slight negative pressure inside the culture vessel. This pressure difference resulted in the inflow of air through the aeration port. The average oxygen mass transfer coefficient k_L_a achieved was 110 h^−1^, which allowed DO levels well above 30% saturation throughout the cultivation process. The pH was not monitored online nor controlled during the cultivation, as the medium was a priori adjusted to a pH of 6.0, which resulted in a final pH of 5.5 in the cultivation broth. The pH was measured at-line on a daily basis from the outflow of the reactors and after harvest. The pH remained constant as soon as steady-state was achieved and the reactor effluent showed a minor deviation of 0.1 pH units (data not shown).

### Exploring 3-HP production in small-scale chemostats at different dilution rates under C-limiting conditions

**Fig. 2:**
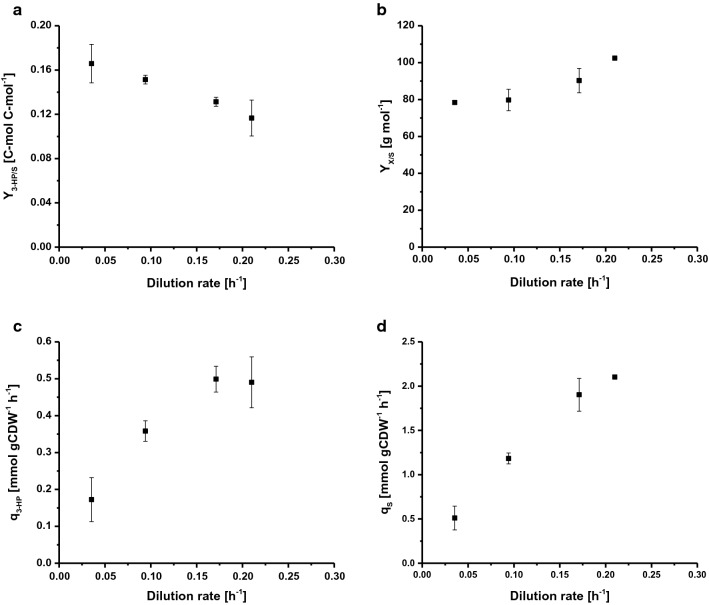
Selected rates and yields for C-limited aerobic chemostat cultivations of *S. cerevisiae* ST938 **a** 3-HP yield (C-mol C-mol^−1^) on glucose, **b** biomass yield on glucose (g mol^−1^), **c** specific 3-HP production rate (mmol gCDW^−1^ h^−1^) and **d** specific glucose uptake rate (mmol gCDW^−1^ h^−1^) at different dilution rates for *S. cerevisiae* ST938. Cultivations were carried out in triplicates at 30 °C and pH 5.5 under C-limited conditions. Errors correspond to standard deviations derived from triplicate cultivations

To determine the maximum specific growth rate (μ_max_), *S. cerevisiae* ST938 was cultivated in batch conditions applying excess nutrient availability. Using glucose as carbon source, the μ_max_ was 0.265 h^−1^, the biomass yield was 24.9 g mol^−1^, and the 3-HP carbon yield on glucose was 0.6 %C-mol (Table 1). In this cultivation mode, most of the carbon was metabolized to ethanol and CO_2_ [9], as high glycolytic fluxes in wild-type *S. cerevisiae* are strongly linked to alcoholic fermentation [10, 11]. Chemostat cultivations are controlled by the supply of a growth-limiting substrate. At steady state, wild-type *S. cerevisiae* does not produce significant quantities of overflow metabolites below a certain growth rate, marking the critical dilution rate (D_crit_ (h^−1^)) due to the lack of accumulation of carbon source. At or above this critical dilution rate carbon source accumulates in the reactor and overflow metabolism is triggered, resulting in the production of various by-products, such as ethanol, acetate and minor quantities of organic acids [9, 11, 12]. Accordingly, continuous cultures were used to investigate the relationship between the growth rate and biomass-specific 3-HP product formation in *S. cerevisiae* ST938. The four different dilutions rates D (h^−1^): 0.04, 0.09, 0.17 and 0.21 were chosen to be below the μ_max_ of *S. cerevisiae* strain ST938 determined in batch cultivation. The biomass yield on glucose as well as the 3-HP yield, specific 3-HP production rate, and the specific substrate uptake rate were investigated (Fig. 2a–d, Table 1). All relevant cultivation parameters, i.e. yields and rates with the respective standard deviations, are summarized in Table 1.

**Table 1:** Cultivation parameters of *S. cerevisiae* ST938 grown in continuous cultures under C-limited conditions

	Chemostat				Batch
	Dilution rate (h^−1^)				µ_max_ (h^−1^)
	0.04	0.09	0.17	0.21	0.27
Y_X/S_ (g mol^−1^)	78.4 ± 0.6	79.7 ± 5.8	90.3 ± 6.6	102.4 ± 0.7	24.9 ± 1.3
Y_EtOH/S_ (mol mol^−1^)	0.09 ± 0.03	0.04 ± 0.02	0.04 ± 0.01	0.04 ± 0.00	1.1 ± 0.0
Y_Gly/S_ (mol mol^−1^)	0.03 ± 0.01	0.02 ± 0.00	0.02 ± 0.00	0.00 ± 0.00	0.1 ± 0.0
Y_3-HP/S_ (mol mol^−1^)	0.33 ± 0.03	0.30 ± 0.00	0.26 ± 0.00	0.23 ± 0.00	0.01 ± 0.00
Y_3-HP/S_ (%C-mol)	16.6 ± 1.7	15.1 ± 0.4	13.1 ± 0.4	11.7 ± 1.5	0.6 ± 0.0
Y_3-HP/X_ (g gCDW^−1^)	0.43 ± 0.04	0.34 ± 0.03	0.26 ± 0.01	0.19 ± 0.02	0.04 ± 0.00
q_S_ (mmol gCDW^−1^ h^−1^)	0.51 ± 0.13	1.2 ± 0.1	1.9 ± 0.2	2.1 ± 0.0	10.7 ± 0.7
q_Gly_ (mmol gCDW^−1^ h^−1^)	0.01 ± 0.00	0.02 ± 0.00	0.03 ± 0.00	0.06 ± 0.01	0.86 ± 0.03
q_EtOH_ (mmol gCDW^−1^ h^−1^)	0.05 ± 0.02	0.05 ± 0.02	0.07 ± 0.02	0.08 ± 0.01	11.9 ± 0.5
q_3-HP_ (mmol gCDW^−1^ h^−1^)	0.17 ± 0.06	0.36 ± 0.03	0.50 ± 0.03	0.49 ± 0.07	0.12 ± 0.00
Glc_res_ (mM)	0.07 ± 0.00	0.08 ± 0.01	0.06 ± 0.03	0.05 ± 0.02	n.a.

Errors correspond to standard deviations derived from triplicate cultivations

*Y* yield, *q* rate, *X* biomass, *S* substrate (glucose), *EtOH* ethanol, *Gly* glycerin, *3-HP* 3-hydroxypropionic acid, *Glc*_*res*_ residual glucose, *n.a.* not applicable

The cultivation of *S. cerevisiae* ST938 under C-limiting conditions showed a switch from predominantly fermentative metabolism observed in batch mode to a respiratory metabolism in chemostats, which is reflected in higher biomass yields as well as in negligible ethanol and glycerol formation (Table 1). Furthermore, minor quantities of residual glucose below 0.1 mM were detected in the samples taken from the efflux of the different reactors, verifying the cultures to be glucose-limited. Carbon-limited conditions found in steady-state chemostat cultures seemed to promote the formation of 3-HP, as the yields of this product were 20- to 25-fold higher than the yield determined in batch culture (Table 1). This is most likely due to the more efficient conversion of carbon into energy in form of ATP during respiration in comparison to fermentation in batch cultures. Interestingly, the cultivations of *S. cerevisiae* ST938 under C-limiting conditions revealed that with declining specific growth rates a constant increase in 3-HP carbon yields could be observed, with the highest 3-HP yield of 16.6 %C-mol observed at a dilution rate of 0.04 h^−1^ (Fig. 2a). The 3-HP yield on biomass is tenfold higher than the yield in batch cultures and increased more than twofold from 0.19 to 0.43 g gCDW^−1^ by lowering the dilution rate from 0.21 to 0.04 h^−1^ (Table 1). The maximum specific productivity for 3-HP of approximately 0.50 mmol gCDW^−1^ h^−1^ was seen at dilution rates of 0.17 and 0.21 h^−1^, which is roughly fourfold higher than measured in batch cultivations (Fig. 2c, Table 1). As expected, the specific glucose uptake rate (q_S_) increased with higher dilution rates from 0.51 to 2.1 mmol gCDW^−1^ h^−1^ (Fig. 2d). These values are up to 20-fold below the maximum specific glucose uptake rate of 10.7 mmol gCDW^−1^ h^−1^ observed at µ_max_ under glucose-limited growth conditions in batch mode (Table 1). Values obtained for the specific substrate uptake rate and biomass yield at a dilution rate of 0.09 h^−1^ (Table 1) were in agreement with data from chemostat cultivations with wild-type *S. cerevisiae* at a dilution rate of 0.10 h^−1^, stating 1.1 and 1.25 mmol gCDW^−1^ h^−1^ as well as 0.49 g g^−1^, respectively [13, 14]. It has been found that the biomass yield per substrate increases with increasing dilution rates, for wild-type *S. cerevisiae*, however, this parameter is generally constant below D_crit_ as long as maintenance metabolism does constitute a considerable carbon sink reducing the biomass yield at low dilution rates [11, 12, 15]. In case of *S. cerevisiae* strain ST938, however, it seemed that carbon, which was not directed towards biomass formation, was contributing to some extent to the formation of both 3-HP and by-products.

To conclude, differences experienced in continuous cultures at different dilution rates as well as in comparison to similar set-ups with *S. cerevisiae* wild-type suggest that the integrated biosynthetic pathway to produce 3-HP has a tremendous impact on yeast physiology, which is presumably amplified by adaptive stress responses. Finally, C-limited chemostat cultivation at the lowest dilution rate of 0.04 h^−1^ resulted in the highest 3-HP carbon yield.

### Investigating nitrogen and phosphate limitation for increased 3-HP yields in small-scale chemostat cultures

After characterizing *S. cerevisiae* ST938 under C-limiting conditions, cultivations under nitrogen- and phosphate- limiting conditions were performed, as they have previously been proven to be favorable for the production of various native and non-native metabolites in different organisms [16–21]. Here, the corresponding cultivations under nitrogen (N) and phosphorus (P) limitation were investigated in the small-scale continuous cultivation system (Fig. 1) with a set dilution rate of 0.04 h^−1^, which had previously resulted in the highest carbon yields of 3-HP under C-limited conditions (Table 1). The parameters and respective standard deviations from N- and P-limited cultivations of yeast strain ST938 are summarized in Table 2.

**Table 2:** Cultivation parameters of *S. cerevisiae* ST938 grown in continuous cultures at a dilution rate of 0.04 h^−1^ under carbon (C), nitrogen (N) and phosphorus (P) limited conditions

	Chemostat [0.04 h^−1^]		
	C-limitation	N-limitation	P-limitation
Y_X/S_ (g mol^−1^)	78.4 ± 0.6	72.4 ± 0.5	75.2 ± 1.9
Y_EtOH/S_ (mol mol^−1^)	0.09 ± 0.03	0.05 ± 0.01	0.04 ± 0.01
Y_Gly/S_ (mol mol^−1^)	0.03 ± 0.01	0.09 ± 0.01	0.01 ± 0.00
Y_3-HP/S_ (mol mol^−1^)	0.33 ± 0.03	0.36 ± 0.04	0.42 ± 0.01
Y_3-HP/S_ (%C-mol)	16.6 ± 1.7	17.7 ± 1.9	21.1 ± 1.8
Y_3-HP/X_ (g gCDW^−1^)	0.43 ± 0.04	0.44 ± 0.05	0.50 ± 0.01
q_S_ (mmol gCDW^−1^ h^−1^)	0.51 ± 0.13	0.61 ± 0.01	0.54 ± 0.01
q_Gly_ (mmol gCDW^−1^ h^−1^)	0.01 ± 0.00	0.06 ± 0.01	0.01 ± 0.00
q_EtOH_ (mmol gCDW^−1^ h^−1^)	0.05 ± 0.02	0.03 ± 0.01	0.02 ± 0.01
q_3-HP_ (mmol gCDW^−1^ h^−1^)	0.17 ± 0.06	0.22 ± 0.02	0.23 ± 0.01
Glc_res_ (mM)	0.07 ± 0.00	0.63 ± 0.13	0.19 ± 0.03
PO_4res_ (mM)	n.a.	n.a.	b.d.
NH_4res_ (mM)	n.a.	b.d.	n.a.

Errors correspond to the standard deviations derived from triplicate cultivations. The C-limitation data were taken from Table 1 for simplicity of comparison

*Y* yield, *q* rate, *X* biomass, *S* substrate (glucose), *EtOH* ethanol, *Gly* glycerin, *3-HP* 3-hydroxypropionic acid, *Glc*_*res*_ residual glucose, *PO*_*4res*_ residual phosphate, *NH*_*4res*_ residual ammonium, *n.a.* not applicable, *n.d.* not determined, *b.d.* below detection limit

For N- and P-limited cultivation conditions, no residual ammonium or phosphate were detected in the samples taken from the efflux of the different reactors, verifying the cultures to be limited with respect to the corresponding substrate limitation (Table 2). However, minor quantities of residual glucose in the range of 0.63 and 0.19 mM were detected under N- and P-limitation (Table 2). During C-limitation the residual glucose levels were well below 0.1 mM (Table 2). Cultivations applying N- and P-limitations revealed a 3-HP yield of 17.7 and 21.1 %C-mol, respectively, displaying a significantly higher value under P-limited conditions compared to the 3-HP carbon yield of 16.6 %C-mol calculated for C-limited cultivation conditions (Tables 1, 2). Similarly, the specific productivity for 3-HP was significantly increased for N- and P-limited cultivations revealing values of 0.22 and 0.23 mmol gCDW^−1^ h^−1^ in comparison to 0.17 mmol gCDW^−1^ h^−1^ reached in C-limited cultures. Moreover, the 3-HP yields on biomass showed comparable values of 0.43 and 0.44 g gCDW^−1^ for C- and N-limiting conditions, however, in case of P-limitation, an increased yield of 0.50 g gCDW^−1^ was observed. Due to the overall low concentrations of by-products quantified, these are negligible (Table 2). The values for the specific substrate uptake rate (q_S_) for C- and P-limitation were comparable and a slightly elevated value of 0.61 mmol gCDW^−1^ h^−1^ was obtained under N-limited conditions. Biomass yields per substrate revealed for N- and P-limited conditions similar values of 72.4 and 75.2 g mol^−1^, respectively. Compared to the biomass yield of 78.4 g mol^−1^ achieved under C-limited conditions, these values are similar. Our study supports the rationale that higher product yields per substrate were achieved under N- and P-limiting conditions. Consequently, it appears that a higher specific substrate uptake rate in case of N-limitation combined with the minimal formation of by-products ultimately favored product formation. Under P-limiting conditions the least amounts of by-products were formed, which could favor product synthesis. It can further be speculated that some metabolic changes possibly lead to a lowered amount of carbon being released as CO_2_.

In summary, chemostat cultivations under N- and P-limitation revealed increased 3-HP yields and specific production rates compared to C-limited conditions, with P-limitation enabling the highest product yields.

### Transferability of the physiological parameters measured in small-scale chemostats to fed-batch cultures in 1-L bench-top bioreactors under C- and P-limiting conditions

**Fig. 3:**
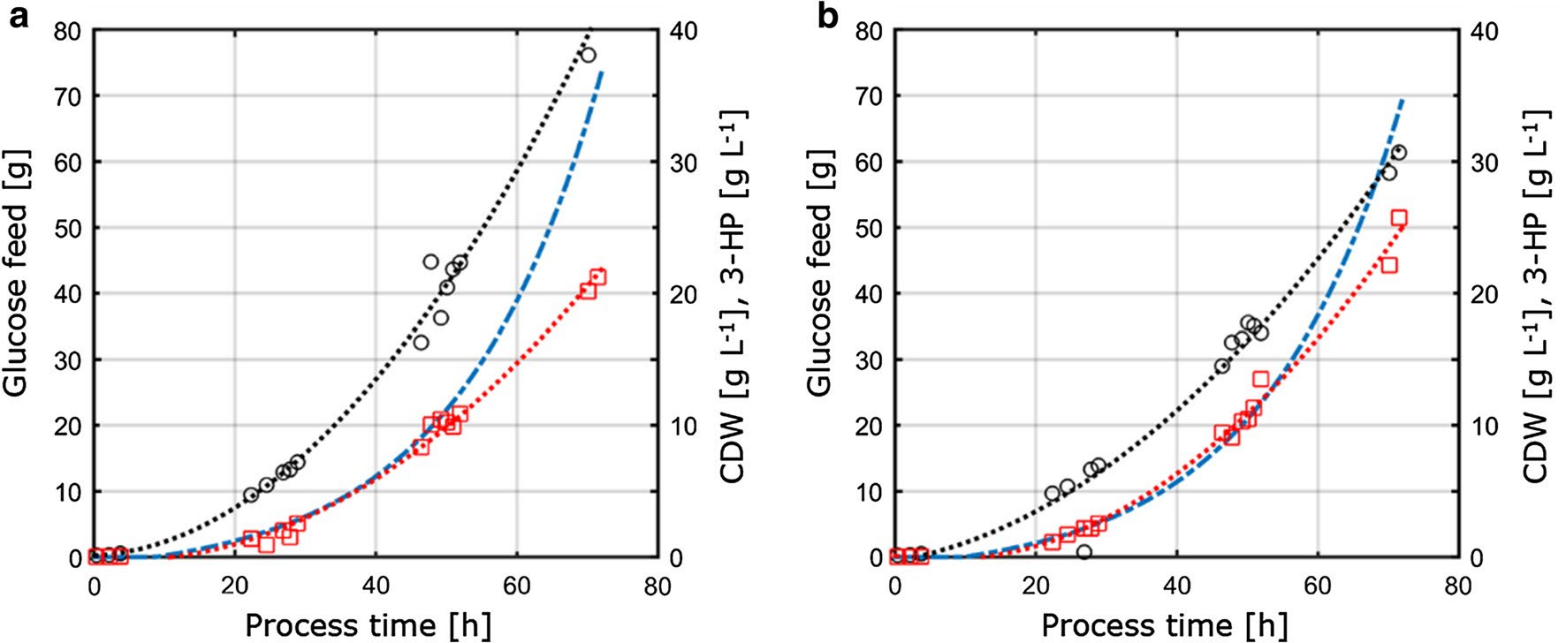
Aerobic fed-batch cultivation in 1-L bench-top fermenters of *S. cerevisiae* ST938 using an exponential feeding ramp at D = 0.05 h^−1^ with the limiting substrate **a** carbon, or **b** phosphorus. Black circles refer to biomass titer, red squares to 3-HP titer and blue dashed line to the absolute amount of glucose fed to the reactors

Since this study further aimed at assessing transferability and comparability of the physiological parameters measured under different cultivation conditions, the cultivation parameters obtained from the C- and P-limiting conditions determined in chemostat cultures were transferred to 1-L stirred bench-top reactors running in fed-batch mode. The concept of this approach was therefore to keep key conditions and parameters constant for chemostat and fed-batch cultures to assure comparability. These conditions comprised (i) the same C:P ratio as applied in P-limiting chemostat cultivations, (ii) the identical process conditions like pH and temperature, and (iii) the same specific growth rates as applied in chemostats, achieved through an exponential feed profile. To optimize the product titer, the parameters with the maximal product yield per substrate from the chemostat experiments (D = 0.04 h^−1^) under C- and P-limitation were chosen for the transfer to the fed-batch system. Due to technical restrictions in the set-up, fed-batch cultivations were carried out at a growth rate of 0.05 h^−1^, which is slightly higher compared to the set dilution rate of 0.04 h^−1^ in chemostat cultivations. The fed-batch cultivation consisted of an initial batch phase to generate biomass followed by an exponential, nutrient limited feeding phase to control the growth rate. Only the feeding phase was considered relevant for the transferability assessment of the parameters obtained from the continuous cultivation system, since solely during this phase the substrate concentration was controlling and thus limiting microbial growth. The fed-batch cultivation profiles of *S. cerevisiae* ST938 applying C- and P-limiting conditions are shown in Fig. 3, and the corresponding cultivation parameters with their respective standard deviation are summarized in Table 3.

**Table 3:** Cultivation parameters of *S. cerevisiae* ST938 grown in fed-batch mode in 1-L bench-top bioreactors under carbon (C) and phosphorus (P) limiting conditions

	Fed-batch	
	C-limitation	P-limitation
M (h^−1^)	0.059	0.051
Y_X/S_ (g mol^−1^)	77.9 ± 8.9	67.7 ± 6.4
Y_EtOH/S_ (mol mol^−1^)	b.d.	b.d.
Y_Gly/S_ (mol mol^−1^)	b.d.	b.d.
Y_3-HP/S_ (%C-mol)	15.9 ± 1.2	25.6 ± 1.7
Y_3-HP/X_ (g gCDW^−1^)	0.38 ± 0.02	0.65 ± 0.09
q_S_ (mmol gCDW^−1^ h^−1^)	0.75 ± 0.10	0.77 ± 0.05
q_3-HP_ (mmol gCDW^−1^ h^−1^)	0.24 ± 0.00	0.39 ± 0.01
q_CO2_ (mmol gCDW^−1^ h^−1^)	1.94 ± 0.48	1.88 ± 0.16
Carbon balance (%)	105 ± 4.3	100 ± 4.2

The growth rate (µ_set_) of the exponential feeding profile was 0.05 h^−1^. Errors correspond to the standard deviations derived from triplicate cultivations

*X* biomass, *S* substrate (glucose), *EtOH* ethanol, *Gly* glycerin, *3-HP* 3-hydroxypropionic acid, *b.d.* below detection limit

**Fig. 4:**
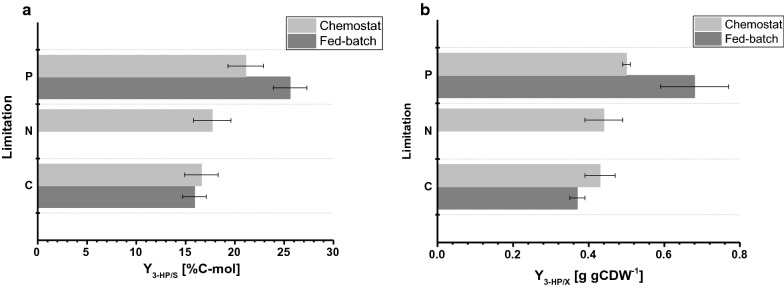
Comparison of cultivation parameters under different limitations determined in small-scale chemostats and 1-L bench-top reactors in fed-batch mode. **a** 3-HP carbon yield (%C-mol) and **b** 3-HP yield on biomass (g gCDW^−1^). Errors correspond to standard deviations derived from triplicate cultivations

The biomass yield observed in the fed-batch cultures was 77.9 and 67.7 g mol^−1^ for C- and P-limitation, respectively. The biomass yield for C-limited cultivations was comparable among chemostat and fed-batch operation, whereas for P-limitation, a slightly decreased biomass yield was observed during fed-batch operation (Tables 2, 3), which is within the standard deviations observed for both biomass yields. The carbon yield per substrate for 3-HP was 15.9% for C-limitation, which is almost identical compared to the value calculated in the chemostat system (Fig. 4a). The 3-HP yields determined in this study are further in close agreement to a carbon yield per substrate of 14% obtained in previous C-limited fed-batch studies at pH 5.0 with a *S. cerevisiae* strain engineered to use the β-alanine pathway [8] and of 13% with a *S. cerevisiae* utilizing the malonyl-CoA reductase-dependent pathway [22]. Higher 3-HP carbon yields per substrate via the β-alanine pathway were determined in studies with *Escherichia coli* stating a value of 42% in fed-batch cultivations on glucose [23].

For P-limiting conditions, however, our study revealed a considerable increase in 3-HP carbon yield of 25.6% (Fig. 4a). The observed 3-HP yields on biomass were 0.38 and 0.65 g gCDW^−1^ for C- and P-limiting conditions, respectively. For C-limitation this confirmed the values observed in the chemostat set-up (Fig. 4b). Due to the increase in yield of 3-HP per substrate under P-limiting conditions and the slight decrease in biomass yield per substrate at the same time, the yield of 3-HP per biomass was significantly increased compared to the chemostat experiment. In contrast to the cultivations carried out in chemostats, no significant accumulation of ethanol or glycerol was detected in fed-batch cultivations. A closed carbon balance for C- and P-limiting conditions indicated that no relevant amounts of other (by-)products were formed (Table 3). This difference in by-product spectrum might explain the increase in 3-HP yield per glucose.

In comparison to the cultivations conducted in chemostats, the specific glucose uptake rates determined in fed-batch cultures show considerably higher values of 0.75 and 0.77 mmol gCDW^−1^ h^−1^ for C- and P-limiting conditions, respectively. As mentioned above, the set point for the exponential factor of the feed profile was set to 0.05 h^−1^ and varied up to 0.059 h^−1^, resulting in a 30–45% higher specific growth rate in fed-batch cultivations compared to the chemostat experiments with 0.04 h^−1^ (Table 3), causing increased specific glucose uptake rates. Nonetheless, for C-limited fermentations, the biomass yield on glucose was comparable between chemostat and fed-batch cultivation. Similarly, the specific 3-HP production rates were elevated in fed-batch cultivations, with 0.24 mmol gCDW^−1^ h^−1^ and 0.38 mmol gCDW^−1^ h^−1^, respectively. 3-HP yields per substrate were comparable between chemostat and fed-batch cultivation, suggesting yields to be a robust cultivation parameter that is resistant to perturbations induced by smaller fluctuations in the cultivation set-up. No residual phosphate, as well as glucose, was detected in the samples taken from the different reactors for P-limiting conditions, confirming the cultures to be limited with respect to the corresponding limitation. Similarly, for C-limitation no residual glucose was detected in the samples.

The here presented improvements of a small-scale chemostat system, previously developed by Klein et al. [2], comprised among others an increase in the number of reactors to 24 and a reduction in cultivation volume to 6.5 mL. This increased degree of parallelization makes the system a suitable high-throughput screening tool, as various comparable small-scale chemostat systems operate with higher working volumes and with a lower number of reactors: The continuous parallel shaken bioreactor (CosBios) system uses six [24] respectively eight parallel culture vessels with a culture volume of 20–25 mL [25]. The for continuous cultivations modified single-use stirred-tank bioreactor system (bioREACTOR, 2mag AG) operates with eight parallel reactors at a working volume of 10 mL [26]. The mini-chemostat (MC) system developed by Bergenholm et al. [27] comprises 16 parallel reactors and requires a working volume of 40 mL. Our system is therefore well applicable for the simple and cost-effective screening of microbial performance in continuous mode. It could be of relevance, however, to further extent the system by monitoring additional parameters, such as exhaust gas, to allow a detailed analysis of the carbon distribution, which is of particular importance for physiological strain characterizations.

As our study suggested N- and P-limitation to be favorable for 3-HP formation in *S. cerevisiae*, future experiments could involve further screening of growth rates with N- and P-limitation applied in order to find the optimum 3-HP production with each respective limitation. Our study further showed the transferability of physiological parameters from chemostat to fed-batch cultivations. This deems only be feasible if no toxic or inhibiting compounds accumulate during the fed-batch cultivation, since physiological parameters would change over time and deviate from the acquired parameters in chemostats. As a general approach, chemostat experiments can serve as a tool for investigating the influence of a potential toxic or inhibiting compound by adding the substance to the feed itself. Since all other parameters are constant, the influence of the compound and its concentration can directly be assessed and evaluated. In our study, there was no by-product formation detected in fed-batch cultivations and a presumably similar stress response was induced due to weak acids, which allowed physiological conditions in small-scale chemostats to resemble to those in fed-batch conditions.

In summary, this study showed that the concept of C- and P-limiting conditions for the production of 3-HP was investigated in a novel parallelized chemostat cultivation system and could successfully be transferred to 1-L bench-top bioreactors operating in fed-batch mode. Therefore, physiological parameters acquired in chemostats can be used for the design and performance assessment of fed-batch cultivations using yield-based parameters for the process set-up.

## Conclusion

This study demonstrates the concept of transferring relevant cultivation parameters from a parallelized small-scale chemostat system to a bench-top fed-batch process to produce 3-hydroxypropionic acid (3-HP) in engineered *S. cerevisiae*, where a suitable growth rate clearly functions as a robust key parameter for process transfer allowing comparable product yields in both systems. The observed physiological performance of engineered *S. cerevisiae* ST938 in chemostats under substrate limiting conditions revealed the highest 3-HP yield from glucose with 21.6% under P-limitation at the lowest growth rate (0.04 h^−1^) tested. To our best knowledge, this is so far the highest yield from carbon achieved with *S. cerevisiae* for 3-HP production as well as the first study that shows that P-limitation has a beneficial effect on 3-HP yield.

Furthermore, it was shown that these parameters can be used for process development in industrially relevant fed-batch applications, as demonstrated in the reproducible production performance of *S. cerevisiae* in fed-batch conditions under C- and P-limitation.

## References

[1] Hemmerich J, Noack S, Wiechert W, Oldiges M (2018). Microbioreactor systems for accelerated bioprocess development. Biotechnol J.

[2] Klein T, Schneider K, Heinzle E (2013). A system of miniaturized stirred bioreactors for parallel continuous cultivation of yeast with online measurement of dissolved oxygen and off-gas. Biotechnol Bioeng.

[3] Kunze M, Lattermann C, Diederichs S, Kroutil W, Büchs J (2014). Minireactor-based high-throughput temperature profiling for the optimization of microbial and enzymatic processes. J Biol Eng.

[4] Hoskisson PA, Hobbs G (2005). Continuous culture—making a comeback?. Microbiology.

[5] Pronk JT, Steensma HY, Van Dijken JP (1996). Pyruvate metabolism in *Saccharomyces cerevisiae*. Yeast.

[6] Hensing MCM, Rouwenhorst RJ, Heijnen JJ, van Dijken JP, Pronk JT (1995). Physiological and technological aspects of large-scale heterologous-protein production with yeasts. Antonie Van Leeuwenhoek.

[7] Lim HC, Chen BJ, Creagan CC (1977). An analysis of extended and exponentially-fed-batch cultures. Biotechnol Bioeng.

[8] Borodina I, Kildegaard KR, Jensen NB, Blicher TH, Maury J, Sherstyk S (2015). Establishing a synthetic pathway for high-level production of 3-hydroxypropionic acid in *Saccharomyces cerevisiae* via β-alanine. Metab Eng.

[9] van Dijken JP, Scheffers WA (1986). Redox balances in the metabolism of sugars by yeasts. FEMS Microbiol Lett.

[10] Verduyn C, Zomerdijk TPL, van Dijken JP, Scheffers WA (1984). Continuous measurement of ethanol production by aerobic yeast suspensions with an enzyme electrode. Appl Microbiol Biotechnol.

[11] Postma E, Verduyn C, Scheffers WA, Van Dijken JP (1989). Enzymic analysis of the crabtree effect in glucose-limited chemostat cultures of *Saccharomyces cerevisiae*. Appl Environ Microbiol.

[12] van Hoek P, van Dijken JP, Pronk JT. Effect of specific growth rate on fermentative capacity of Baker’ s yeast. Appl Environ Microbiol. 1998;64:4226–33. http://aem.asm.org/. 10.1128/aem.64.11.4226-4233.1998 PMC106631 9797269

[13] Siew LT, Boer VM, Daran-Lapujade P, Walsh MC, De Winde JH, Daran JM (2005). Two-dimensional transcriptome analysis in chemostat cultures: combinatorial effects of oxygen availability and macronutrient limitation in *Saccharomyces cerevisiae*. J Biol Chem.

[14] Vemuri GN, Eiteman MA, McEwen JE, Olsson L, Nielsen J (2007). Increasing NADH oxidation reduces overflow metabolism in *Saccharomyces cerevisiae*. Proc Natl Acad Sci.

[15] Rieger M, Käppeli O, Fiechter A (1983). The role of limited respiration in the incomplete oxidation of glucose by *Saccharomyces cerevisiae*. J Gen Microbiol.

[16] Shu P, Johnson MJ (1948). Citric acid production by submerged fermentation with *Aspergillus niger*. Ind Eng Chem.

[17] Derrick S, Large PJ (1993). Activities of the enzymes of the Ehrlich pathway and formation of branched-chain alcohols in *Saccharomyces cerevisiae* and *Candida utilis* grown in continuous culture on valine or ammonium as sole nitrogen source. J Gen Microbiol.

[18] Ding Y, Li S, Dou C, Yu Y, He H (2011). Production of fumaric acid by *Rhizopus oryzae*: role of carbon–nitrogen ratio. Appl Biochem Biotechnol.

[19] Kristiansen B, Sinclair CG (1979). Production of citric acid in submerged culture. Biotechnol Bioeng.

[20] Wu S, Hu C, Jin G, Zhao X, Zhao ZK (2010). Phosphate-limitation mediated lipid production by *Rhodosporidium toruloides*. Bioresour Technol.

[21] Ryu HW, Hahn SK, Chang YK, Chang HN (1997). Production of poly(3-hydroxybutyrate) by high cell density fed-batch culture of *Alcaligenes eutrophus* with phosphate limitation. Biotechnol Bioeng.

[22] Kildegaard KR, Jensen NB, Schneider K, Czarnotta E, Özdemir E, Klein T (2016). Engineering and systems-level analysis of *Saccharomyces cerevisiae* for production of 3-hydroxypropionic acid via malonyl-CoA reductase-dependent pathway. Microb Cell Fact.

[23] Song CW, Kim JW, Cho IJ, Lee SY (2016). Metabolic engineering of *Escherichia coli* for the production of 3-hydroxypropionic acid and malonic acid through β-alanine route. ACS Synth Biol.

[24] Akgün A, Müller C, Engmann R, Büchs J (2008). Application of an improved continuous parallel shaken bioreactor system for three microbial model systems. Bioprocess Biosyst Eng.

[25] Sieben M, Steinhorn G, Müller C, Fuchs S, Ann Chin L, Regestein L (2016). Testing plasmid stability of *Escherichia coli* using the Continuously Operated Shaken BIOreactor System. Biotechnol Prog.

[26] Schmideder A, Severin TS, Cremer JH, Weuster-Botz D (2015). A novel milliliter-scale chemostat system for parallel cultivation of microorganisms in stirred-tank bioreactors. J Biotechnol.

[27] Bergenholm D, Liu G, Hansson D, Nielsen J (2019). Construction of mini-chemostats for high-throughput strain characterization. Biotechnol Bioeng.

